# Study on the Effect of Residual Polymer Superplasticizer on the Properties of Graphene–Cement Composites

**DOI:** 10.3390/polym16070956

**Published:** 2024-03-31

**Authors:** Ki Yun Kim, Seok Hwan An, Jea Uk Lee

**Affiliations:** Department of Advanced Materials Engineering for Information and Electronics, Integrated Education Institute for Frontier Science and Technology (BK21 Four), Kyung Hee University, 1732 Deogyeong-daero, Giheung-gu, Yongin-si 17104, Gyeonggi-do, Republic of Korea; 12rldbs@khu.ac.kr (K.Y.K.); ansh0703@khu.ac.kr (S.H.A.)

**Keywords:** graphene, superplasticizer, polycarboxylate ether, centrifugation, cement nanocomposites, compressive strength

## Abstract

Graphene, renowned for its exceptional mechanical, thermal, and electrical properties, is being explored as a cement nanofiller in the construction field. However, the limited water dispersibility of graphene requires the use of polymer superplasticizers, such as polycarboxylate ether (PCE). Previous studies have investigated the mechanisms by which PCE facilitates the dispersion of graphene within cement nanocomposites. However, such studies have made minimal progress, indicating a lack of understanding of the effect of residual PCE (rPCE) remaining in aqueous solution without binding to graphene. In this study, the effects of rPCE on the dispersion of graphene and the mechanical properties of graphene–cement composites (GCCs) were systematically analyzed. For this purpose, the content of rPCE was accurately measured through the centrifugation process and thermal analysis of graphene dispersion with PCE, and the result was 78.0 wt.% compared to graphene. The optical microscopy, particle size analysis, and contact angle measurement of the graphene dispersions with and without rPCE confirmed that rPCE is crucial for the dispersion of graphene and the enhancement of the interfacial affinity between graphene and cement. Additionally, the compressive strength of GCC with rPCE exhibited a substantial enhancement of approximately 10% (68.36 MPa) compared to plain cement (62.33 MPa). The effectiveness of rPCE in enhancing compressive strength correlated with the uniform dispersion of graphene within GCC and the promotion of cement hydration, as evidenced by field emission scanning electron microscopy and X-ray diffraction, respectively.

## 1. Introduction

Cement, recognized as one of the most popular construction materials globally, has been the subject of extensive research due to its inherent mechanical strength limitations. For over half a century, advancements in nanotechnology driven by numerous studies have explored the use of silica fume or fly ash to enhance the mechanical strength of cement [1,2]. Nano-silica, characterized by a spherical shape with a diameter of under 30 nm and a specific surface area of 300 m^2^/g, has been widely employed for the development of high-strength cement composites by promoting the cement hydration and microstructure densification [3,4]. However, the low aspect ratio of the nano-silica hinders its ability to withstand micro-cracks caused by nano-sized fissures, thereby limiting its reinforcing effect and, thus, the applicability in the cement composites [5].

In recent years, to overcome these shortcomings of nano-silica, there has been a growing interest in research focusing on the incorporation of one-dimensional (1D) carbon nanomaterials such as carbon nanofibers (CNFs) and carbon nanotubes (CNTs) into cement. Carbon-based nanomaterials, renowned for their superior mechanical properties and high aspect ratio, have demonstrated a significant potential in delaying microcrack formation, reducing porosity, and promoting hydration in cement [6,7,8,9]. Similarly, graphene, a two-dimensional (2D) carbon nanomaterial comprising sp^2^-bonded carbon atoms, exhibits remarkable mechanical properties, including an intrinsic strength ranging from 60 to 130 GPa and a Young’s modulus of over 1 TPa [10,11]. In addition, the graphene features a flat sheet with a thickness of only one atom layer, and a larger specific surface area compared to other carbon nanomaterials [12,13,14,15,16,17,18]. It has been reported that the graphene sheets promote hydration of the cement by providing nucleation sites for the formation of the hydration products, such as portlandite and C-S-H gel. Furthermore, their nanofiller effect decreases the porosity of the cement by filling and blocking the coarse pores [19,20]. 

Graphene is broadly classified into three types, graphene oxide (GO), reduced graphene oxide (rGO), and exfoliated graphene, depending on the manufacturing method and content of functional groups on its basal plane and edges. Graphene oxide is easily dispersed in water thanks to its abundant hydrophilic functional groups, but it has inferior physical properties compared to other forms of graphene. Reduced graphene oxide, which recovers the defects of graphene oxide, has the issue of significantly lower water dispersibility. Exfoliated graphene exhibits significantly superior mechanical properties compared to chemically synthesized graphenes, such as GO and rGO. However, the hydrophobic surface of the exfoliated graphene hinders its dispersion in water and causes agglomeration issues within the graphene-reinforced cement composite (GCC) [21,22,23].

Hence, ensuring the homogeneous dispersion of exfoliated graphene within the GCC is imperative to maximize its advantageous properties. While numerous studies have employed surfactants to enhance the dispersion of the graphene in polymer composites, this strategy could potentially lead to adverse effects on the properties of GCC due to side reactions between the surfactants and cement [24]. Therefore, recent studies have focused on controlling the aggregation of the exfoliated graphene in GCC by utilizing superplasticizers, which effectively decrease the water content per unit of cement [25]. Although negatively charged lignosulphonates, water-soluble anionic polyelectrolyte polymers, and naphthalene-based superplasticizers have been employed as dispersing agents based on an electrostatic repulsion mechanism, they prove insufficient in attaining a uniform and stable dispersion of the exfoliated graphene in GCC [25]. Recently, a comb-shaped polymer structure of polycarboxylate ether (PCE), comprising a hydrophilic charged polymethacrylic backbone and a less hydrophilic polyethylene glycol side chain, has attracted considerable attention as a dispersing agent for GCC [25,26,27]. The backbone of the PCE imparts hydrophilicity, while the side chain forms van der Waals interactions with the graphene surface, creating a steric hindrance that prevents graphene aggregation.

Although substantial research has been conducted on the combination of graphene and PCE and its influence on the mechanical properties of GCC, there has been minimal progress in analyzing how residual PCE (rPCE), not combined with graphene, affects the microstructure and resulting mechanical properties of GCC. In this study, to understand the impact of rPCE within GCC, a centrifugation process was used to prepare a graphene dispersion sample without rPCE, which was then compared with a sample containing rPCE. An optical microscope and particle size analyzer were employed to determine the influence of rPCE on the dispersion of pristine graphene. The contact angle measurement was utilized to observe whether rPCE improves the surface affinity between graphene and cement. X-ray diffraction was measured to determine the effect of rPCE on the hydration of GCC. To observe the microstructure of the GCC, field-emission scanning electron microscopy was employed. Finally, a compressive strength test of GCC was conducted according to the rPCE content.

## 2. Experimental Section

### 2.1. Materials

Type 1 Ordinary Portland Cement (Asia Cement, Co., Ltd., Seoul, Republic of Korea) was used to prepare cement paste with graphene, and 1 wt.% graphene aqueous dispersion was supplied from Elves Chemical Co., Ltd. (Anseong-si, Gyeonggi-do, Republic of Korea). PCE (WD500, KG chemical, Co., Ltd., Ulju, Republic of Korea) was utilized as a polymer superplasticizer for the dispersion of the pristine graphene.

### 2.2. Dispersions of Graphene/PCE

**Figure 1:**
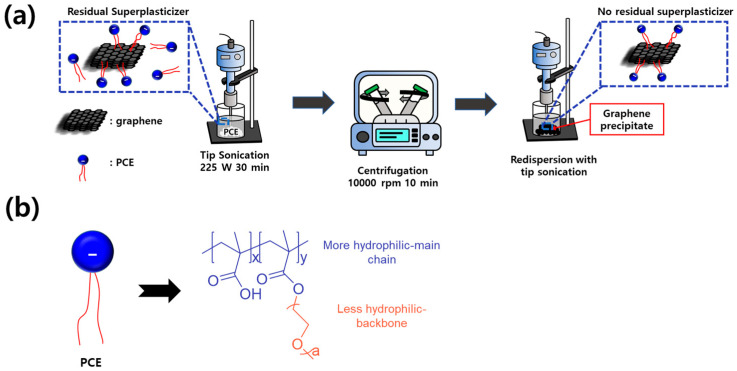
(**a**) Production procedure for G/PCE 1:1, G/PCE C1, and G/PCE C2. (**b**) Chemical structure of PCE.

The manufacturing procedure, along with the presence of the residual superplasticizer, is illustrated in Figure 1a. To prepare a pristine graphene dispersion with PCE, PCE powder was added to a 1 wt.% graphene aqueous solution in a 1:1 ratio of graphene and PCE. To achieve a homogeneous dispersion of graphene, the tip sonicator (ULH 700S, ULSSO Hi-Tech, Co. Ltd., Cheongju, Republic of Korea) was utilized, applying 225 W of ultrasonic energy for 30 min, with a cycle of 5 s of energy application, followed by a 5 s rest. The resulting dispersion was labeled G/PCE 1:1. To assess the effect of rPCE and facilitate a comparison, centrifugation (1248, Labogene, Lillerød, Denmark) was employed. The G/PCE 1:1 underwent centrifugation at 10,000 rpm for 10 min, followed by the collection and subsequent drying of the precipitate in a convection oven at 70 °C for 24 h, yielding a G/PCE hybrid material. The ratio of graphene to PCE in the solid was verified through a thermogravimetric analysis (TGA, SDT Q600, TA Instruments, New Castle, DE, USA). Following this, a 1 wt.% graphene–PCE hybrid dispersion was prepared using the G/PCE hybrid under the same conditions, with 1 h of tip sonication. This dispersion was labeled G/PCE C1. Similarly, the G/PCE C2 was prepared using the same process involving centrifugation, drying, and tip sonication. 

Figure 1b represents the chemical structure of PCE superplasiticizer. PCE, characterized by a comb-shaped polymer structure, comprises a more hydrophilic charged polymethacrylic backbone and a less hydrophilic polyethylene glycol side chain. The side chains of PCE form van der Waals interactions with graphene, while the more hydrophilic backbone enhances affinity with water [27]. The degree of dispersibility in the three samples (G/PCE 1:1, G/PCE C1, and G/PCE C2) was assessed using an optical microscope (OM, BX43-P, OLYMPUS, Tokyo, Japan) for qualitative analysis and a particle size analyzer (PSA, Mastersizer 3000E, Malvern Panalytic, Malvern, UK) for quantitative analysis.

### 2.3. Contact Angle Measurement

Contact angle measurements were performed to assess the interfacial affinity improvement provided by rPCE between graphene and cement. The cement-to-water ratio (W/C) was fixed at 0.3, and the cement paste was produced using a planetary paddle mixer. After casting the paste into a mold measuring 50 mm × 50 mm × 20 mm, it was allowed to hydrate for 28 days, resulting in the formation of a cement plate. The average contact angle between the dispersion and the cement plate was determined by repeatedly dropping 1.5 μL G/PCE droplets onto the cement surface, which was accomplished using the Phoenix 300 (SEO Co., Ltd., Suwon, Republic of Korea). Previous research has established that a lower contact angle between the G/PCE dispersion and the cement plate corresponds to a higher interfacial affinity [28].

### 2.4. Compressive Strength Measurement

**Figure 2:**
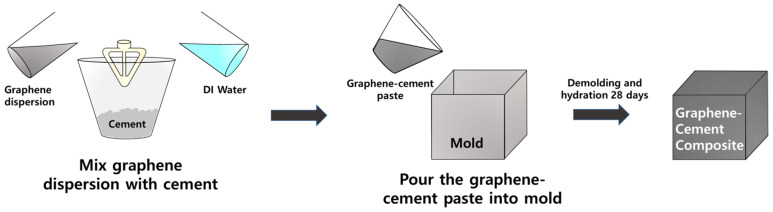
Manufacturing process of graphene–cement composite.

The GCC samples with pristine graphene, G/PCE 1:1, G/PCE C1, and G/PCE C2 were consistently prepared with a fixed graphene content of 0.01 wt.% relative to the cement. The cement-to-water ratio (W/C) was maintained at 0.3. In total, five types of samples were produced, including plain cement. The detailed ratios are outlined in Table 1, and the PCE content in G/PCE C1 and G/PCE C2 was determined through TGA. The GCC manufacturing process is illustrated in detail in Figure 2. The cement was mixed according to the ratio outlined in Table 1, using a planetary paddle mixer, and then cast into molds (50 mm × 50 mm × 50 mm). The top of the mold was sealed with plastic wrap to prevent moisture evaporation from the sample, and it was stored under standard laboratory conditions for a day. Subsequently, the samples were removed from the molds and submerged in a saturated lime solution at a controlled temperature of 23 ± 2 °C for 28 days. Thereafter, the sample was removed from the lime solution and dried for 3 h at room humidity. Compressive strength tests were conducted following ASTM C 109 standards, using a digital electrical compressive strength tester (HS-1472, Hanshin Kumpoong. Co., Ltd., Gimpo, Republic of Korea). The compressive strength test was conducted until the sample reached the fracture point.

### 2.5. Microstructure Analysis

After measuring the compressive strength, the degree of hydration in GCC was determined by observing the presence of crystalline hydrates, specifically portlandite, using X-ray diffraction (XRD) to study the effect of the presence of rPCE on the quantity of hydrate. XRD measurements were performed with a Miniflex 300 Desktop X-Ray diffractometer (Rigaku, Tokyo, Japan) at 30 kV/15 mA, employing CuKα radiation (λ = 1.5418 Å). Furthermore, the distribution and morphology of graphene and hydrates in GCC were examined using field-emission scanning electron microscopy (SEM, LEO SUPRA 55, Carl Zeiss, Oberkochen, Germany).

## 3. Results and Discussion

### 3.1. Thermogravimetric Analysis

**Figure 3:**
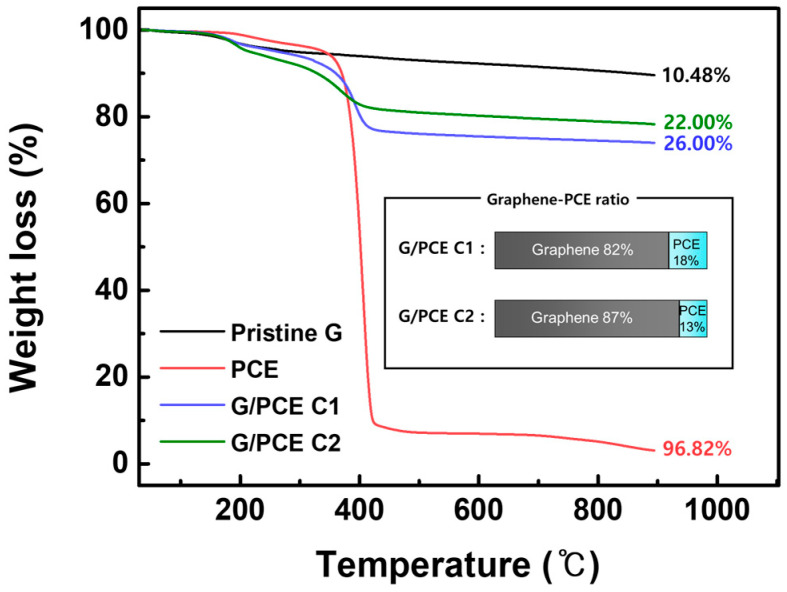
TGA graphs of pristine graphene, pure PCE, G/PCE C1, and G/PCE C2.

Figure 3 depicts the TGA results for the pristine graphene (labeled by Pristine G in the TGA graph), PCE, G/PCE C1, and G/PCE C2. TGA was conducted with a heating rate of 10 °C/min, within the temperature range from 35 °C to 900 °C. The PCE content in G/PCE C1 and G/PCE C2 was quantified by comparing the weight loss (%) of pristine graphene and PCE. The calculation was performed using the following equation:

$$\frac{X_{G}\%}{100}\times \left(100-Y_{P}\right)+\frac{X_{P}\%}{100}\times Y_{P}=X_{G/P}\%$$

where *X_G_*, *X_P_*, and *X*_*G*/*P*_ are the weight loss of graphene, PCE, and G/PCE C1 or C2, respectively; and *Y_P_* is the mass ratio of PCE to the total hybrid.

Pristine graphene exhibited a minor mass loss of approximately 10.48% up to 900 °C, attributed to its high crystallinity and low oxygen content. On the other hand, since the backbone and graft part of PCE are both composed of flexible linear polymer, a significant mass portion of PCE was decreased at 900 °C. Thus, the mass reduction of the hybrid sample comprising graphene and PCE (G/PCE) can primarily be attributed to the content of PCE. In the case of G/PCE C1 (mass loss of 26.0%), 22.0 wt.% of PCE compared to graphene remained within the hybrid material, representing a physically bound fraction [29]. Similarly, G/PCE C2 (mass loss of 22.0%) retained 15.0 wt.% of PCE compared to graphene, also in a physically bound state. As the centrifugation progressed, the physical bonds between PCE and graphene were disrupted, resulting in a decrease in the PCE content. Therefore, it was confirmed that the 78.0 wt.% of residual PCE (rPCE) could be completely removed via the repetitive centrifugation process of the dispersion of graphene and PCE. We performed a similar experiment using sodium naphthalene formaldehyde (SNF), a commonly used superplasticizer in cements like PCE. The chemical structure and TGA analysis of the hybrid sample comprising graphene and SNF (G/SNF) are shown in Supplementary Figure S1. Unlike the use of PCE, it was confirmed through TGA that all SNFs were detached from graphene with just one centrifugation, as evidenced by the overlap of the pristine graphene and G/SNF C1 graph. This suggests that PCE exhibits a higher affinity with graphene compared to SNF.

### 3.2. Optical Microscope Analysis

**Figure 4:**
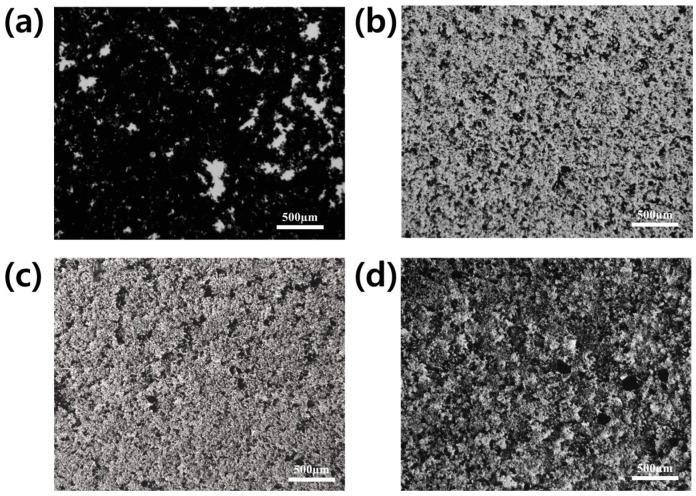
OM images of (**a**) pristine graphene, (**b**) G/PCE 1:1, (**c**) G/PCE C1, and (**d**) G/PCE C2 dispersions.

Figure 4 displays OM images of pristine graphene, G/PCE 1:1, G/PCE C1, and G/PCE C2 aqueous dispersions. Pristine graphene (Figure 4a) tends to agglomerate in DI water due to the absence of hydrophilic functional groups on its surface. As a result, due to the strong interactions among themselves through van der Waals forces [21], pristine graphene was not uniformly dispersed in water and agglomerated. On the other hand, due to the effect of PCE as a dispersing agent, the G/PCE 1:1 sample showed very uniform dispersion from a macroscopic perspective (Figure 4b). As reported in the previous studies [26], PCE exhibits amphiphilic characteristics, enabling it to function as an effective dispersing agent alongside its role as a polymer superplasticizer for cement. This resulted in the homogeneous dispersion of graphene nanoplatelets in the water.

In contrast, the G/PCE C1 dispersion demonstrated diminished dispersibility, which can be attributed to a lower PCE content compared to G/PCE 1:1 (Figure 4c) [25]. The G/PCE 1:1 sample contained 100 wt.% PCE relative to graphene, whereas, in the G/PCE C1 sample, a significant portion of the residual PCE was removed through the centrifugation process, resulting in only 22.0 wt.% PCE remaining (Figure 3). Lastly, G/PCE C2 exhibited the lowest dispersibility due to its lower PCE content (15.0 wt.%) compared to the other samples (Figure 4d). Therefore, it was found that both the PCE physically bound to graphene and the residual PCE are crucial for the dispersion of graphene in water. In summary, G/PCE 1:1, distinguished by its highest PCE content, including rPCE (78.0 wt.%), displayed minimal aggregation and a more uniform distribution, primarily attributable to the steric hindrance effect of PCE.

### 3.3. Particle Size Analysis

**Figure 5:**
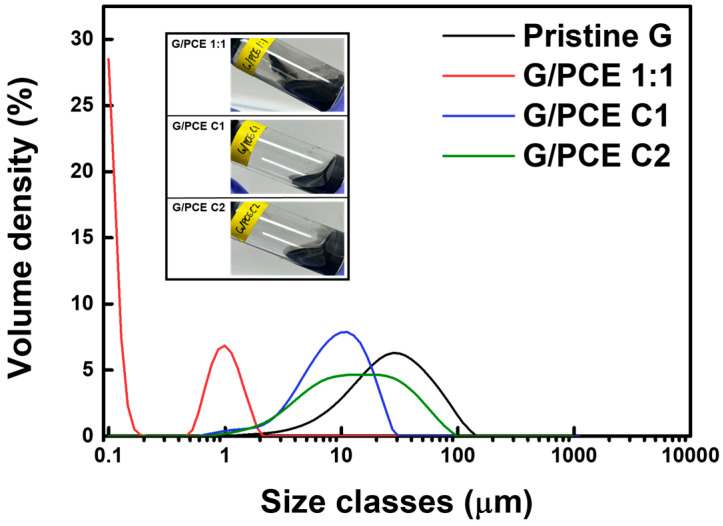
Particle size analysis of pristine graphene, G/PCE 1:1, G/PCE C1, and G/PCE C2 dispersions.

A particle size analysis was employed for the quantitative analysis of the dispersion degree of graphene in water, leveraging the fact that a higher specific surface area, lower average area of particles (D (3,2)), and smaller particle size (Dv (50)) correspond to better dispersion [30]. As shown in Figure 5 and Table 2, pristine graphene exhibited a minimum specific surface area of 333.1 m^2^/kg and maximum D (3,2) and Dv (50) values of 18.0 µm and 28.2 µm, respectively, in the aqueous dispersion due to agglomeration driven by van der Waals forces [21]. In contrast, G/PCE 1:1 displayed significantly lower D (3,2) and Dv (50) values of 0.19 µm and 0.14 µm. This phenomenon was attributed to the presence of excess residual PCE in the G/PCE 1:1 dispersion, which is observed in the 0.1-to-0.2 µm region of the particle size distribution graph [31,32].

On the other hand, it can be observed that the pure PCE was not detected in the 0.1–0.2 µm region of the particle size distribution of G/PCE C1 and C2, confirming that the rPCE was completely removed from the G/PCE samples through repeated centrifugation processes. As a direct consequence of the reduced PCE content, G/PCE C1 exhibited increased D (3,2) and Dv (50) values of 6.61 µm and 9.42 µm, respectively, while G/PCE C2 had 8.56 µm and 14.00 µm, respectively. Through these data, it was confirmed again that the presence of rPCE is crucial for the dispersion of graphene in aqueous dispersion [33]. Furthermore, the broad particle size distribution of the G/PCE C2 sample (green curve of the graph) suggests that the dispersion of graphene became highly non-uniform due to the absence of rPCE and the decrease in physically bound PCE on the surface of graphene.

### 3.4. Contact Angle Measurements

**Figure 6:**
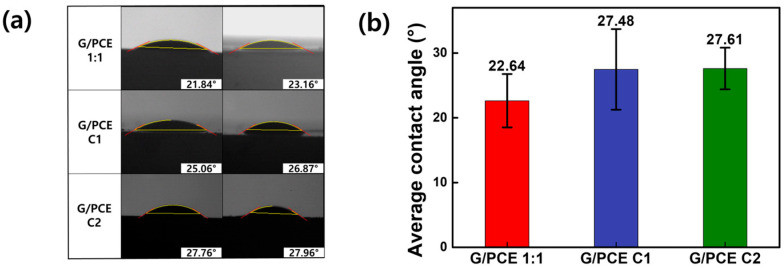
(**a**) Photo images of contact angle measurement of G/PCE 1:1, G/PCE C1, and G/PCE C2 dispersions with cement plate. (**b**) Average contact angles.

Our previous studies confirmed that the measurement of the contact angle between graphene dispersions and cement plates serves as an indicator of the interfacial affinity between the two materials and the effect of rPCE [28]. As a consequence of the lack of hydrophilic functional groups on the surface of pristine graphene, the formation of an aqueous dispersion was unattainable, thus precluding the measurement of the contact angle with cement plate (Supplementary Figure S2) [1,20]. Figure 6a provides visual representations of the contact angles of the three kinds of graphene dispersion (G/PCE 1:1, G/PCE C1, and G/PCE C2), while Figure 6b exhibits the average contact angle of these dispersions. In the case of the G/PCE 1:1 dispersion, the contact angle with the cement plate was notably lower (22.64°) than that of the G/PCE C1 (27.48°) and G/PCE C2 (27.61°) dispersions. Recognizing that a lower contact angle signifies a higher interfacial affinity between graphene dispersions and cement plate, the observed low contact angle of G/PCE 1:1 suggests that rPCE effectively contributes to improving the interfacial affinity between graphene and cement [28]. This phenomenon can be interpreted in two ways. First, since PCE is widely utilized as a superplasticizer for cement, it can improve the affinity between the aqueous solution and cement material. The second rationale is that PCE functions as an amphiphilic surfactant, thereby augmenting the affinity of hydrophobic graphene with water and hydrophilic cement plate. In conclusion, the presence of rPCE demonstrated its effectiveness in enhancing both the dispersion of graphene in aqueous solution and its affinity with cement.

### 3.5. Compressive Strength Test

**Figure 7:**
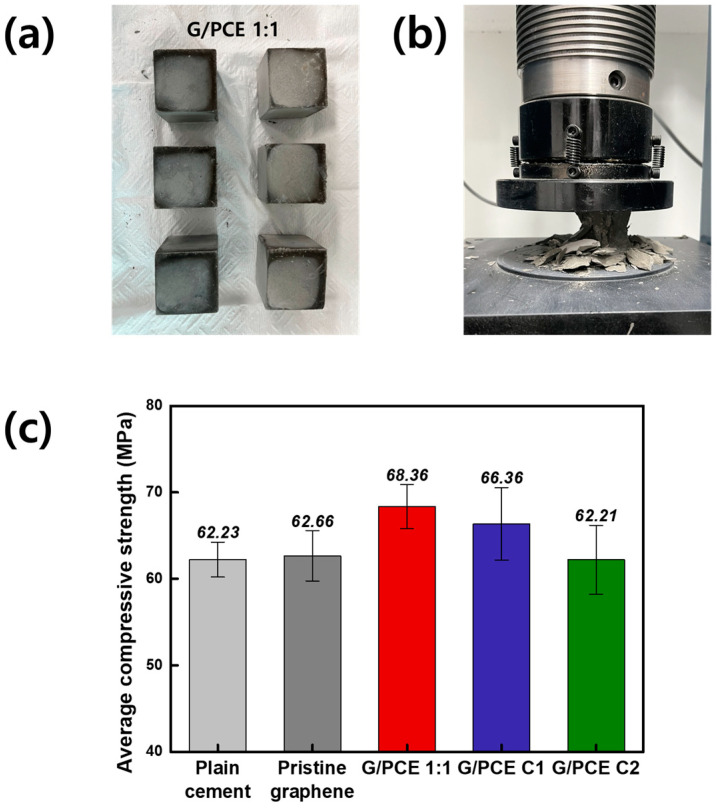
Photographs of (**a**) GCC samples made from G/PCE 1:1 dispersion and (**b**) compressive strength test. (**c**) Average compressive strength of GCC samples made from plain cement, pristine graphene, G/PCE 1:1, G/PCE C1, and G/PCE C2 dispersions.

To assess the impact of rPCE in graphene dispersion on the strength of GCC, the compressive strength of cement composites produced from each graphene dispersion was measured. Figure 7a,b show photographs of the fabricated GCC samples through water curing for 28 days and the process of conducting the compressive strength test, respectively. Specifically, Figure 7a depicts GCC samples made from the G/PCE 1:1 dispersion, while other sample images of plain cement and GCCs from pristine graphene, G/PCE C1, and G/PCE C2 dispersions are provided in Supplementary Figure S3. Figure 7c displays the average compressive strengths of the plane cement and GCC samples, derived from the measurement of six cement cubes. The compressive strength of the GCC sample with only pristine graphene added was recorded at 62.66 MPa, which was very similar to the average value observed for the plain cement specimen without graphene (62.23 MPa). The GCC sample prepared exclusively with pristine graphene without PCE is interpreted to lack a reinforcing effect, attributed to the severe agglomeration of graphene during the cement manufacturing process (Figure 4a).

Among the samples, the GCC prepared from the G/PCE 1:1 dispersion, distinguished for its exceptional dispersibility observed through the OM and particle size analysis, demonstrated the highest compressive strength, i.e., 68.36 MPa. This indicates approximately a 10% enhancement in the compressive strength compared to the plain cement sample. The improved strength can be attributed to the uniform dispersion of graphene within the cement, which facilitates cement hydration by providing nucleation sites of graphene and reduces porosity through the filler effect [21]. These assertions will be further substantiated through subsequent XRD and SEM analyses.

The compressive strength of the GCC sample prepared from the G/PCE C1 dispersion was measured at 66.36 MPa, indicating a 6.6% increase compared to the plain cement sample. The diminished strength of the GCC made from G/PCE C1 in comparison to G/PCE 1:1 is interpreted as resulting from a reduced interfacial affinity between the graphene and cement due to the removal of rPCE. The GCC sample made from G/PCE C2 recorded compressive strengths of 62.21 MPa, which were nearly identical to both plain cement and GCC sample containing only pristine graphene. The GCC sample from G/PCE C2 dispersion, in which the PCE on the graphene surface was removed through two centrifugation steps, did not exhibit significant differences compared to the GCC with pristine graphene. These results align with previous research findings, which suggest that the agglomerated graphene within the cement has an adverse effect on compressive strength, or the reinforcing effect may be insignificant [34].

### 3.6. XRD Analysis of GCC

**Figure 8:**
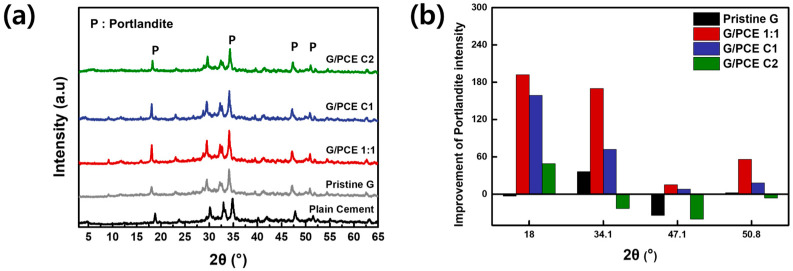
(**a**) XRD analysis of plain cement and GCCs with PCE and (**b**) peak enhancement degree of portlandite peaks of GCCs compared to that of plain cement.

XRD was utilized to quantify the amount of crystalline hydrate contributing to the compressive strength increase in the GCC [35,36]. Figure 8a,b display distinct XRD peaks and the degree of enhancement of the calcium hydroxide peak, identified as portlandite (18°, 34.1°, 47.1°, and 50.8°, marked as ‘P’ on the graph) compared to that of plain cement, respectively [32,37]. Upon scanning the peaks of Figure 8a, it becomes apparent that no discernible new peaks emerged, and the positions of the existing peaks remained unchanged in the cement nanocomposites featuring graphene and PCE. This observation suggests that the presence of graphene sheets or PCE did not result in the formation of any new components during the hydration processes of the cement nanocomposite. However, the GCC, derived from the G/PCE 1:1 dispersion containing rPCE, exhibited a significantly higher intensity at all four portlandite peaks compared to the other cement composites. In particular, except for the peak at 47.1°, the peak intensity of the G/PCE 1:1 sample was increased by more than 50 degrees compared to plain cement. This indicates that the uniform dispersion of graphene with PCE provides nucleation sites for the formation of crystalline hydrate. Furthermore, the existence of rPCE within GCC also plays a role in promoting the hydration of cement [21,25].

GCC, made from the G/PCE C1 dispersion without rPCE, demonstrated the second highest portlandite peak intensity. PCE, physically bonded to the graphene surface, aids in dispersing graphene within cement and offers some nucleation sites for cement hydrate. On the other side, GCCs prepared from pristine graphene and G/PCE C2 dispersion exhibited portlandite peak intensity almost identical to that of plain cement. The limited dispersion of graphene in GCCs from pristine graphene and G/PCE C2 dispersion leads to a delay in the hydration reaction of cement, thereby reducing the peak intensities of portlandite. The XRD intensity corresponds to the quantity of crystalline cement hydrate generated, which decreases the cement’s porosity, thereby enhancing its mechanical strength. The observed difference in XRD intensity among the five cement composites aligns with the compressive strength variations shown in Figure 7c, supporting the fact that cement hydration significantly influences compressive strength [19,35,36].

### 3.7. FE-SEM Analysis of GCC

**Figure 9:**
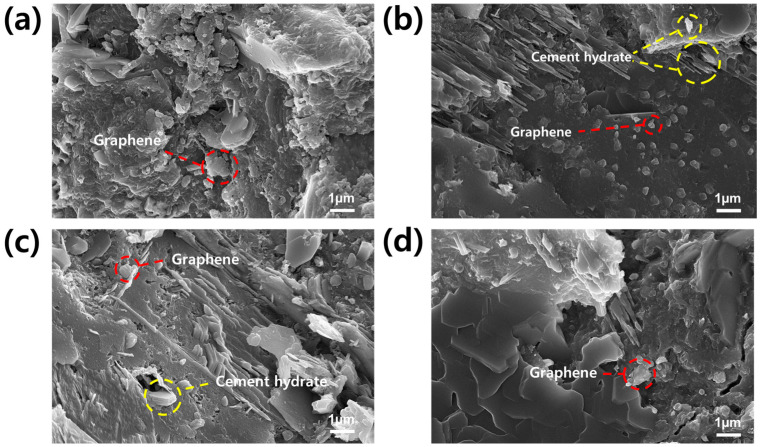
FE-SEM images of GCCs made from (**a**) pristine graphene, (**b**) G/PCE 1:1, (**c**) G/PCE C1, and (**d**) G/PCE C2 dispersions.

Figure 9 shows the distribution of graphene and cement hydrates within the GCC. In Figure 9a, the GCC prepared from pristine graphene without PCE reveals the amorphously grown cement structure accompanied by agglomerated graphene nanoplatelets. On the other hand, Figure 9b, representing GCC made with G/PCE 1:1, exhibits the presence of cement hydrates, alongside evenly dispersed graphene [21]. As demonstrated in Figure 9c, it was confirmed that the cement hydrate, such as portlandite, and dispersed graphene were mixed inside the GCC produced from G/PCE C1. The size of graphene within GCC from G/PCE C1 was observed to be smaller than that of pristine graphene and G/PCE C2 but larger than G/PCE 1:1. GCC prepared from G/PCE C2 (Figure 9d) contained graphene of a size similar to that of pristine graphene sample (Figure 9a). FE-SEM images confirmed that the degree of dispersion and particle size of graphene in GCC were directly influenced by the dispersibility in the aqueous solution (Figure 4 and Figure 5). G/PCE 1:1, characterized by the highest level of dispersion in water, demonstrated the most uniform graphene dispersion within cement, whereas pristine graphene and G/PCE C2 samples exhibited the least dispersion in both the aqueous solution and cement nanocomposites, attributed to the lack of PCE. Furthermore, it was observed that, alongside the dispersion of graphene nanoplatelets, the proliferation of cement hydrates was most pronounced in the GGC derived from the G/PCE 1:1 sample. This finding aligned with the XRD results depicted in Figure 8.

Through FE-SEM observation, the impact of rPCE in the G/PCE 1:1 sample on the cement composites was reaffirmed. rPCE improved the dispersion of graphene in the aqueous solution during the cement manufacturing process, consequently ensuring the uniform dispersion of graphene within the GCC. Additionally, rPCE, in conjunction with dispersed graphene, contributed to promoting cement hydration. Lastly, the presence of rPCE improved the interfacial affinity between the graphene dispersion and cement. These combined factors significantly enhanced the compressive strength of the GCC.

## 4. Conclusions

This study investigated the effects of rPCE on graphene dispersion, the interfacial affinity between graphene and cement, and the resulting properties of the prepared GCC. G/PCE 1:1 dispersion containing a significant amount of rPCE (78.0 wt.% compared to graphene), along with samples from which rPCE was removed (G/PCE C1 and C2), were prepared through a centrifugation process of an aqueous graphene dispersion with PCE. The content of rPCE was quantified through a TGA analysis. Through optical microscopy and particle size analyses of graphene dispersions, it was identified that rPCE enhanced the dispersion of graphene in water. The graphene aqueous dispersion with rPCE exhibited a significantly lower contact angle with the cement plate than other graphene dispersions without rPCE, indicating that rPCE also improved the interfacial affinity between graphene and cement. This improved affinity was further validated using FE-SEM, demonstrating that rPCE not only increased the dispersibility of graphene in water but also ensured the uniform dispersion of graphene in cement within the GCC. The uniformly dispersed graphene in GCC served as a reinforcing agent in cement by providing the nucleation sites, thereby promoting cement hydration. This was confirmed by XRD analyses of GCCs. Consequently, the compressive strength of GCC made with rPCE increased by 10% compared to plain cement. This study demonstrates, for the first time, that rPCE improves graphene dispersion uniformity and cement hydration, which, in turn, increases the compressive strength of GCC. Cement nanocomposites with the rPCE system are expected to offer a facile method for evaluating the dispersion of nanomaterials within cement and their effect on the mechanical properties of cement nanocomposites.

## Figures and Tables

**Table 1:** Mixing proportions of graphene–cement composites.

	W/C (%)	Graphene Content (wt.% to Cement)	Cement (g)	D.I. Water (g)	Water in Graphene Dispersion (g)	Addition of D.I. Water (g)	Graphene (g)	PCE (g)
Plain	0.3	-	1400	420	-	-	-	-
Pristine graphene	0.3	0.01	1400	420	13.86	406.14	0.14	-
G/PCE 1:1	0.3	0.01	1400	420	13.86	406.14	0.14	0.14
G/PCE C1	0.3	0.01	1400	420	13.86	406.14	0.14	0.03
G/PCE C2	0.3	0.01	1400	420	13.86	406.14	0.14	0.02

**Table 2:** The results of particle size analysis.

	Specific Surface Area (m^2^/kg)	D (3,2) (μm)	Dv (50) (μm)
Pristine Graphene	333.1	18.0	28.2
G/PCE 1:1	32,110	0.187	0.136
G/PCE C1	908	6.61	9.42
G/PCE C2	701	8.56	14

D (3,2), average area of particles; Dv (50), corresponding particle size when the cumulative percentage reaches 50%.

## Data Availability

Data are contained within the article and Supplementary Materials.

## Supplementary Materials

The following supporting information can be downloaded at https://www.mdpi.com/article/10.3390/polym16070956/s1, Figure S1: (a) Chemical structure of SNF; and (b) TGA graphs of pristine graphene, pure SNF, and G/SNF C1; Figure S2: Photo image of contact angle measurement of pristine graphene aqueous dispersion with cement plate; Figure S3: Photographs of (a) plain cement and GCC samples made from (b) pristine graphene, (c) G/PCE C1, and (d) G/PCE C2 dispersions.
