# Supplementary material for: Study on the Effect of Residual Polymer Superplasticizer on the Properties of Graphene–Cement Composites
Source: Polymers (Basel). 2024 Mar 31;16(7):956. doi: 10.3390/polym16070956 (PMC11013087; doi:10.3390/polym16070956)
Supplement: Supplementary file 1 [file polymers-16-00956-s001.zip › polymers-2934505-supplementary.pdf]

polymers

Supplementary data

Study on the Effect of Residual Polymer Superplasticizer on the Properties of Graphene–Cement Composites

Ki Yun Kim, Seok Hwan An and Jea Uk Lee *

Department of Advanced Materials Engineering for Information and Electronics, Integrated Education Institute for Frontier Science and Technology (BK21 Four), Kyung Hee University, 1732 Deogyeong-daero, Giheung-gu, Yongin-si 17104, Gyeonggi-do, Republic of Korea; 12rldbs@khu.ac.kr (K.Y.K.); ansh0703@khu.ac.kr (S.H.A.)

* Correspondence: leeju@khu.ac.kr; Tel.: +82-31-201-3655

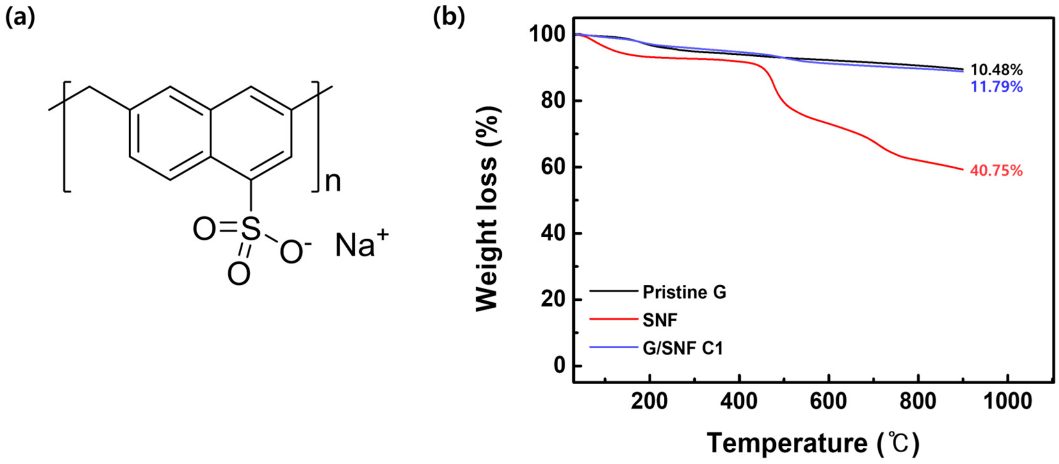

Figure S1. (a) Chemical structure of SNF. (b) TGA graphs of pristine graphene, pure SNF, and G/SNF C1.

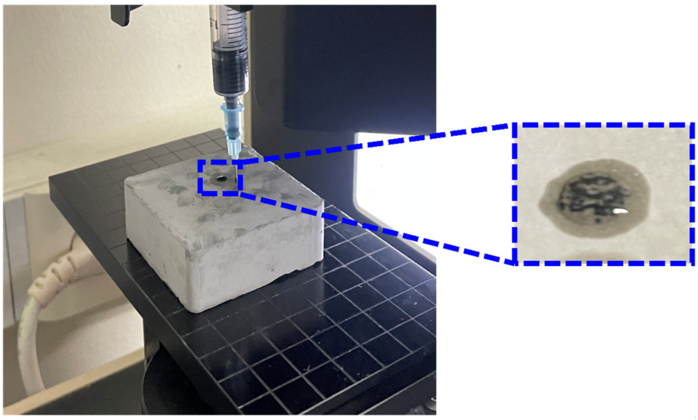

Figure S2. Photo image of contact angle measurement of pristine graphene aqueous dispersion with cement plate.

Citation: Kim, K.Y.; An, S.H.; Lee, J.U. Study on the Effect of Residual Polymer Superplasticizer on the Properties of Graphene–Cement Composites. *Polymers* **2024**, *16*, 956. https://doi.org/10.3390/polym16070956

Academic Editors: Quantao Liu, Wei Du, Yongbin Yan and Xiaobin Han

Received: 11 March 2024
Revised: 28 March 2024
Accepted: 29 March 2024
Published: 31 March 2024

Copyright: © 2024 by the authors. Licensee MDPI, Basel, Switzerland. This article is an open access article distributed under the terms and conditions of the Creative Commons Attribution (CC BY) license (https://creativecommons.org/licenses/by/4.0/).

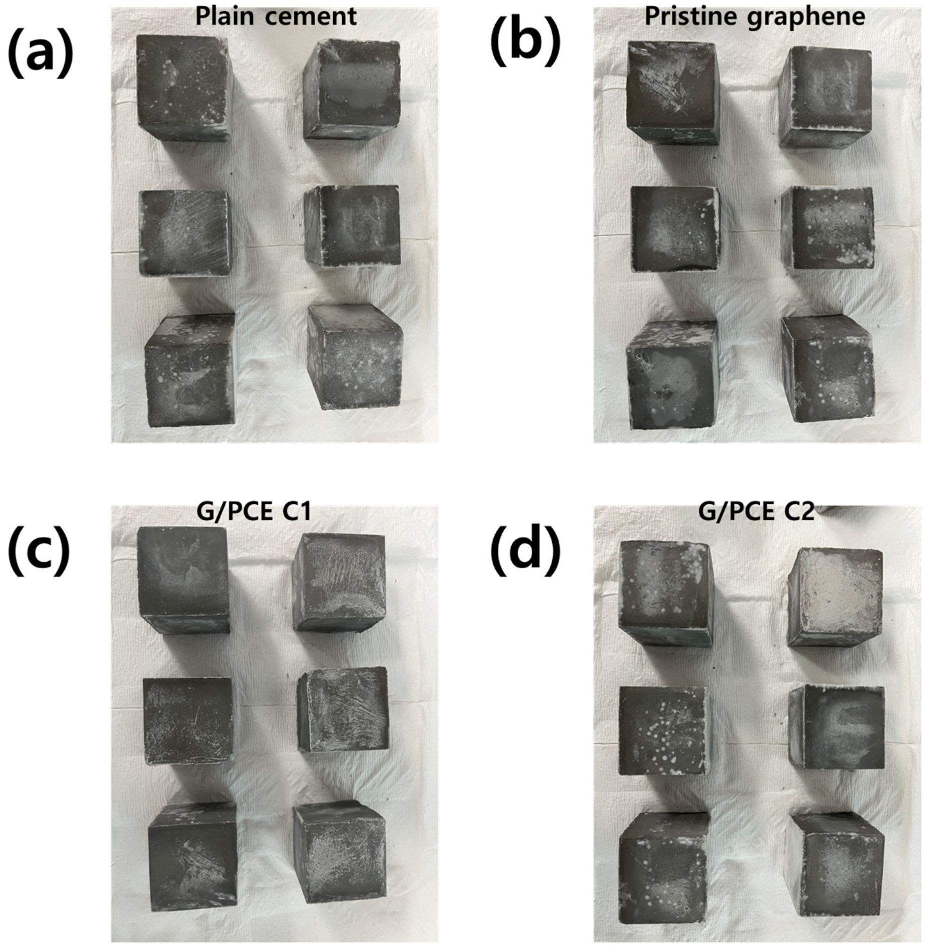

Figure S3. Photographs of (a) plain cement and GCC samples made from (b) pristine graphene, (c) G/PCE C1, and (d) G/PCE C2 dispersions.
